# Supplementary material for: A bedform phase diagram for dense granular currents
Source: Nat Commun. 2020 Jun 8;11:2873. doi: 10.1038/s41467-020-16657-z (PMC7280512; doi:10.1038/s41467-020-16657-z)
Supplement: Supplementary file 1 — Supplementary Information [file 41467_2020_16657_MOESM1_ESM.pdf]

Supplementary Information for **A bedform phase diagram for dense granular currents** by Smith et al.

Supplementary Figures

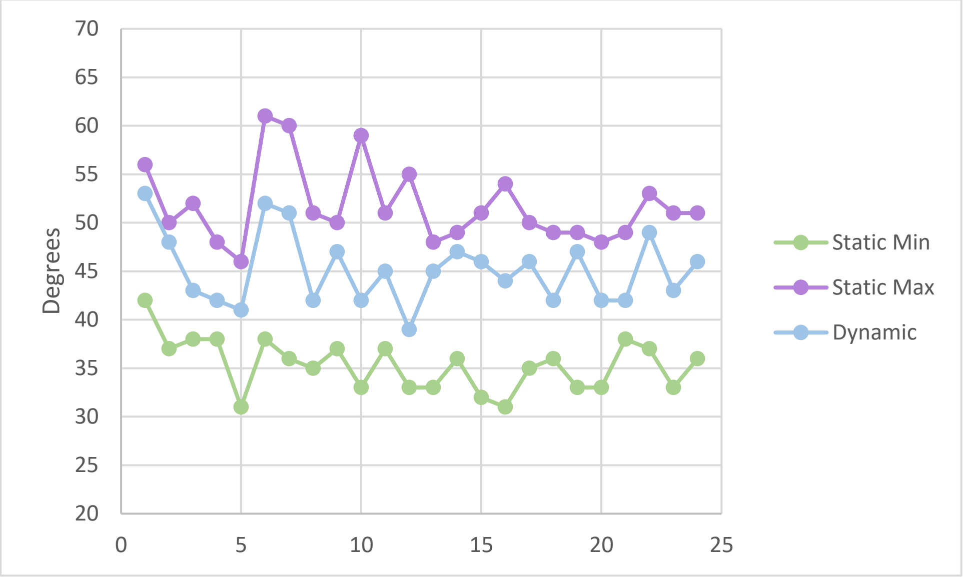

Supplementary Figure 1. Results of rotating drum tests on Pozzolane Rosse ignimbrite samples. Dynamic, maximum and minimum static repose angles are given. X axis is test number.

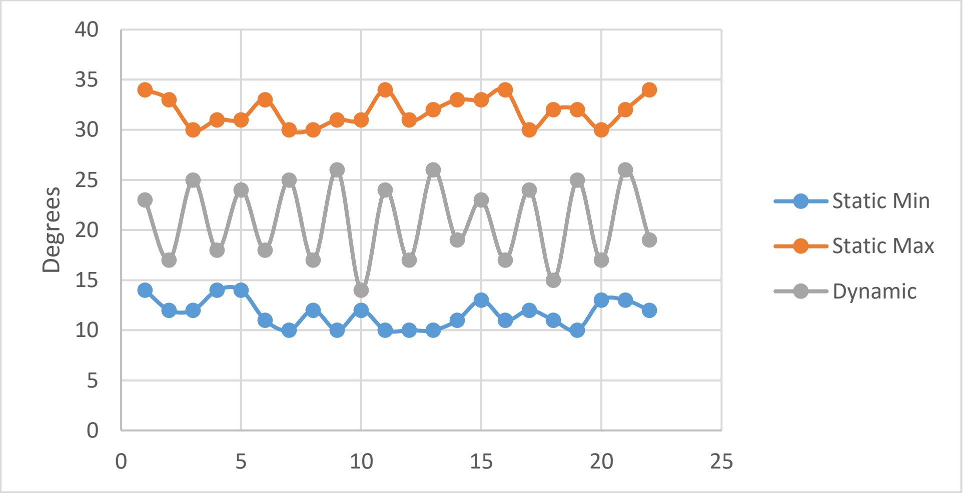

Supplementary Figure 2. Results of rotating drum tests on ballotini samples. Dynamic, maximum and minimum static repose angles are given. X axis is test number.

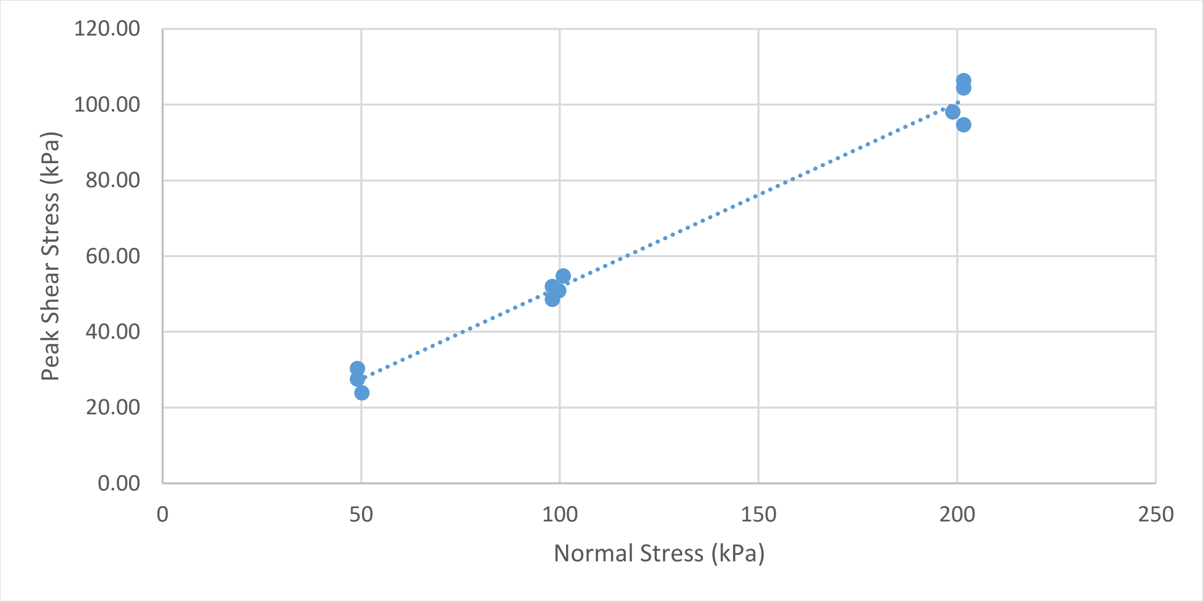

Supplementary Figure 3. Results of shearbox testing on ballotini samples. Cohesion value given by intersect with y-axis. Internal friction angle given by value of slope.

Supplementary Tables

Supplementary Table 1. Experimental current data for five runs.

Run	Bedform	Current velocity	Current thickness	Froude Number	Savage number	Bagnold Number	Friction Number
1	Steep Regressive	0.44	0.0081	1.6	0.0003	41.6	132552
		0.36	0.0060	1.5	0.0005	46.0	88893
		0.22	0.0059	0.9	0.0002	28.6	140654
		0.40	0.0090	1.3	0.0002	33.7	181828
		0.35	0.0075	1.3	0.0002	35.4	144308
		0.33	0.0110	1.0	0.0001	23.0	325943
		0.40	0.0107	1.2	0.0001	28.4	257005
		0.36	0.0102	1.1	0.0001	27.0	256902
		0.44	0.0062	1.8	0.0007	54.4	77661
		0.33	0.0078	1.2	0.0002	31.6	168089
	Shallow Regressive	0.75	0.0120	2.2	0.0003	47.4	172399
		0.73	0.0093	2.4	0.0006	59.8	105901
		0.73	0.0078	2.7	0.0010	71.3	74494
		0.80	0.0075	2.9	0.0013	80.9	63135
		0.50	0.0068	1.9	0.0007	55.8	83039
		0.50	0.0070	1.9	0.0006	54.2	87996
		0.60	0.0053	2.6	0.0020	85.9	42037
		0.53	0.0047	2.5	0.0023	86.1	37190
		0.45	0.0043	2.2	0.0022	79.4	36894
		0.33	0.0047	1.5	0.0009	52.4	61031
		0.95	0.0100	3.0	0.0008	72.1	94517
		0.63	0.0079	2.3	0.0007	60.8	88482
		0.70	0.0050	3.2	0.0033	106.2	32068
		0.70	0.0043	3.4	0.0052	123.5	23718
		0.63	0.0054	2.8	0.0022	89.0	41342
		0.80	0.0070	3.1	0.0016	86.7	54997
		0.44	0.0062	1.8	0.0007	54.4	77661
		0.90	0.0078	3.3	0.0014	87.5	60699
		0.80	0.0071	3.0	0.0015	85.5	56580
		0.80	0.0068	3.1	0.0017	89.2	51899
		0.76	0.0068	2.9	0.0016	84.8	54631
		0.80	0.0075	2.9	0.0013	80.9	63135
		0.67	0.0065	2.6	0.0014	77.8	56905
		0.67	0.0064	2.7	0.0014	79.0	55168
		0.50	0.0060	2.1	0.0010	63.2	64650
	Planar	1.00	0.0057	4.2	0.0046	133.1	29173
		1.27	0.0043	6.2	0.0170	223.4	13107
		0.90	0.0050	4.1	0.0055	136.5	24942
		1.05	0.0048	4.8	0.0084	165.9	19703
		0.80	0.0043	3.9	0.0068	141.1	20753

		1.10	0.0031	6.3	0.0343	269.1	7845
		1.10	0.0036	5.9	0.0219	231.7	10579
		0.88	0.0032	5.0	0.0200	208.6	10448
		0.63	0.0031	3.6	0.0112	153.8	13728
		0.95	0.0040	4.8	0.0119	180.1	15123
		1.05	0.0057	4.4	0.0050	139.7	27784
		1.33	0.0064	5.3	0.0057	158.0	27584
		1.00	0.0069	3.8	0.0026	109.9	42750
		1.00	0.0055	4.3	0.0051	137.9	27162
		0.95	0.0057	4.0	0.0041	126.4	30709
		0.90	0.0038	4.7	0.0125	179.6	14407
2	Steep Regressive	0.63	0.0127	1.8	0.0002	37.8	228670
		0.50	0.0092	1.7	0.0003	41.2	151999
		0.33	0.0072	1.3	0.0003	35.1	139644
		0.67	0.0107	2.1	0.0003	47.3	154203
		0.40	0.0069	1.5	0.0004	44.0	106874
		0.53	0.0099	1.7	0.0002	40.2	167628
		0.33	0.0081	1.2	0.0002	31.2	176736
		0.67	0.0125	1.9	0.0002	40.4	210449
		0.40	0.0127	1.1	0.0001	23.9	362061
		0.25	0.0121	0.7	0.0000	15.7	525854
	Shallow Regressive	1.00	0.0113	3.0	0.0006	66.9	115468
		0.90	0.0091	3.0	0.0009	75.2	82255
		0.73	0.0071	2.8	0.0013	78.3	61723
		0.73	0.0077	2.7	0.0010	72.2	72596
		0.50	0.0062	2.0	0.0009	61.2	69032
		0.67	0.0048	3.1	0.0034	105.3	31032
		0.40	0.0038	2.1	0.0025	79.8	32415
		0.95	0.0065	3.8	0.0028	110.8	39934
		0.70	0.0051	3.1	0.0031	104.1	33364
		0.67	0.0044	3.2	0.0044	114.9	26075
		0.73	0.0045	3.5	0.0050	123.6	24795
		0.36	0.0041	1.8	0.0016	67.3	41508
		0.63	0.0055	2.7	0.0020	86.2	43459
		0.80	0.0051	3.6	0.0041	119.0	29193
		0.67	0.0035	3.6	0.0088	144.5	16499
		0.70	0.0047	3.3	0.0040	113.0	28336
		0.95	0.0059	3.9	0.0037	122.1	32901
		0.55	0.0049	2.5	0.0022	85.1	39198
		0.67	0.0045	3.2	0.0041	112.4	27274
	Planar	1.67	0.0090	5.6	0.0032	140.4	43639
		1.25	0.0083	4.4	0.0023	114.2	49486
		1.25	0.0089	4.2	0.0019	106.5	56899
		1.50	0.0084	5.2	0.0032	135.4	42238
		1.25	0.0082	4.4	0.0024	115.6	48301
		1.47	0.0082	5.2	0.0033	135.7	41165

		1.10	0.0078	4.0	0.0022	107.0	49663
		1.50	0.0110	4.6	0.0014	103.4	72432
		1.25	0.0100	4.0	0.0013	94.8	71833
		1.00	0.0082	3.5	0.0015	92.5	60376
		0.95	0.0080	3.4	0.0015	90.1	60491
		1.25	0.0071	4.7	0.0037	133.5	36211
		1.25	0.0074	4.6	0.0033	128.1	39336
		1.10	0.0067	4.3	0.0034	124.5	36643
		1.20	0.0071	4.5	0.0034	128.2	37720
3	Steep Regressive	0.67	0.0125	1.9	0.0002	40.4	210449
		0.67	0.0094	2.2	0.0005	53.8	119009
		0.53	0.0099	1.7	0.0002	40.2	167628
		0.35	0.0095	1.1	0.0001	27.9	231533
		0.70	0.0145	1.9	0.0001	36.6	269695
		0.45	0.0117	1.3	0.0001	29.2	273145
		0.45	0.0109	1.4	0.0001	31.3	237069
		0.50	0.0123	1.4	0.0001	30.8	271691
		0.42	0.0092	1.4	0.0002	34.3	182399
	Shallow Regressive	0.73	0.0110	2.2	0.0003	50.6	148156
		1.00	0.0100	3.2	0.0008	75.8	89791
		0.88	0.0096	2.9	0.0007	69.5	94036
		0.50	0.0085	1.7	0.0003	44.6	129749
		1.00	0.0076	3.7	0.0019	99.8	51863
		0.90	0.0075	3.3	0.0016	91.0	56120
		1.00	0.0075	3.7	0.0020	101.1	50508
		0.80	0.0078	2.9	0.0012	78.3	67414
		0.80	0.0070	3.1	0.0016	86.7	54997
		0.67	0.0087	2.3	0.0006	58.1	101945
		0.80	0.0091	2.7	0.0007	66.7	92945
	Planar	1.47	0.0083	5.1	0.0032	134.0	42175
		1.40	0.0067	5.5	0.0055	158.5	28791
		1.00	0.0073	3.7	0.0022	103.9	47850
		1.47	0.0070	5.6	0.0053	158.9	29998
		0.90	0.0064	3.6	0.0026	106.7	40865
		1.05	0.0061	4.3	0.0041	130.5	31820
		1.33	0.0100	4.3	0.0015	101.1	67344
		1.00	0.0081	3.5	0.0016	93.6	58912
		1.00	0.0090	3.4	0.0012	84.3	72731
		1.05	0.0073	3.9	0.0024	109.1	45571
		0.90	0.0073	3.4	0.0018	93.5	53166
4	Steep Regressive	0.31	0.0103	1.0	0.0001	22.7	309594
		0.33	0.0067	1.3	0.0003	37.7	120922
	Shallo w Regre	0.67	0.0089	2.3	0.0005	56.8	106686
		0.50	0.0083	1.8	0.0004	45.7	123715

		0.73	0.0092	2.4	0.0006	60.5	103636
		0.80	0.0084	2.8	0.0009	72.2	79196
	Planar	0.80	0.0091	2.7	0.0007	66.7	92945
		1.00	0.0070	3.8	0.0025	108.3	43998
		1.05	0.0081	3.7	0.0018	98.3	56107
5	Shallow Regressive	0.67	0.0094	2.2	0.0005	53.8	119009
		0.73	0.0089	2.5	0.0006	62.5	96987
		0.50	0.0080	1.8	0.0004	47.4	114933
		0.44	0.0066	1.7	0.0006	51.1	88005
	Planar	1.10	0.0074	4.1	0.0025	112.7	44700
		1.20	0.0068	4.6	0.0039	133.8	34600
		1.05	0.0071	4.0	0.0026	112.2	43108
		1.00	0.0065	4.0	0.0031	116.7	37937
		1.05	0.0070	4.0	0.0027	113.8	41903
		1.33	0.0067	5.2	0.0050	150.9	30231

Supplementary Table 2. Grain size data for samples from the Pozzolane Rosse ignimbrite.

	Fraction (mm)	Mass (g)	Proportion	Cumulative Percentage	Fraction (phi)
Sample A	>5.6	13.40	0.40	100.00%	-5.00
	2-5.6	12.45	0.37	59.89%	-2.49
	600-2	4.38	0.13	22.63%	-1.00
	300-600	1.19	0.04	9.52%	0.74
	150-300	0.94	0.03	5.96%	1.74
	63-150	0.75	0.02	3.15%	2.74
	<63	0.30	0.01	0.90%	3.99
		**SUM**		0	7.97
		**33.41**			
Sample B	>5.6	11.80	0.33	100.00%	-5.00
	2-5.6	9.99	0.28	67.12%	-2.49
	600-2	6.39	0.18	39.28%	-1.00
	300-600	2.60	0.07	21.47%	0.74
	150-300	2.17	0.06	14.21%	1.74
	63-150	1.92	0.05	8.18%	2.74
	<63	1.02	0.03	2.83%	3.99
		**SUM**		0	7.97
		**35.89**			
Sample C	>5.6	17.60	0.60	100.00%	-5.00
	2-5.6	4.04	0.14	39.67%	-2.49
	600-2	3.21	0.11	25.83%	-1.00
	300-600	1.38	0.05	14.82%	0.74
	150-300	1.23	0.04	10.09%	1.74
	63-150	1.21	0.04	5.87%	2.74
	<63	0.51	0.02	1.74%	3.99
		**SUM**		0	7.97
		**29.17**			
Sample D	>5.6	8.50	0.28	100.00%	-5.00
	2-5.6	10.78	0.36	71.71%	-2.49
	600-2	5.54	0.18	35.84%	-1.00
	300-600	1.96	0.07	17.40%	0.74
	150-300	1.49	0.05	10.89%	1.74
	63-150	1.26	0.04	5.95%	2.74
	<63	0.53	0.02	1.75%	3.99
		**SUM**		0	7.97
		**30.05**			
Sample E	>5.6	2.60	0.09	100.00%	-5.00
	2-5.6	10.99	0.39	90.84%	-2.49
	600-2	7.71	0.27	52.12%	-1.00
	300-600	2.53	0.09	24.98%	0.74
	150-300	1.85	0.07	16.07%	1.74

	63-150	1.66	0.06	9.56%	2.74
	<63	1.06	0.04	3.72%	3.99
		**SUM**		0	7.97
		**28.39**			
Sample F	>5.6	7.50	0.30	100.00%	-5.00
	2-5.6	8.97	0.36	69.91%	-2.49
	600-2	4.76	0.19	33.91%	-1.00
	300-600	1.79	0.07	14.82%	0.74
	150-300	1.10	0.04	7.66%	1.74
	63-150	0.66	0.03	3.26%	2.74
	<63	0.16	0.01	0.63%	3.99
		**SUM**		0	7.97
		**24.93**			

Supplementary Table 3. Supplementary mechanical data for ballotini.

	50kPa				100kPa			200kPa		
	**Test 1**	**Test 2**	**Test 3**	**Test 4**	**Test 1**	**Test 2**	**Test 3**	**Test 1**	**Test 2**	**Test 3**
**Sample Height (mm)**	3.87	3.21	4.44	8.13	4.89	6.35	3.15	3.32	5.85	4.86
	3.44	3.81	6.2	4.99	5.55	6.46	4.78	6.15	2.01	2.92
	3.40	3.2	5.21	4.06	3.61	6.4	6.54	2.87	5.76	7.09
	4.23	3.26	5.76	2.74	2.9	4.29	9.37	4.87	6.39	5.76
**Av. Sample Height (mm)**	3.74	3.37	5.40	4.98	4.24	5.88	5.96	4.30	5.00	5.16
**Consolidation (mm)**	0.392	0.2	0.204	0.227	0.665	0.51	0.404	0.821	0.637	0.746
**Mass (kg)**	1.84	1.8	1.8	1.8	3.66	3.6	3.6	7.3	7.4	7.4
**Force (N)**	18.05	17.66	17.66	17.66	35.90	35.32	35.32	71.61	72.59	72.59
**Shear rate (mm/min)**	0.5	0.5	0.5	0.5	0.5	0.5	0.5	0.5	0.5	0.5
**Area (m^2)**	0.0036	0.0036	0.0036	0.0036	0.0036	0.0036	0.0036	0.0036	0.0036	0.0036
**Load (N/m^2)**	5014	4905	4905	4905	9973.5	9810	9810	19892.5	20165	20165
**Lever effect**	10	10	10	10	10	10	10	10	10	10
**Normal Stress (kPa)**	50.14	49.05	49.05	49.05	99.735	98.1	98.1	198.925	201.65	201.65
**Peak Shear Force (N)**	86	109	99	97	183	175	187	353	383	341
**Shear Stress (kPa)**	23.89	30.28	27.50	26.94	50.83	48.61	51.94	98.06	106.39	94.72
**Pre-consolidation Volume (mm^3)**	78804	80118	72801	74322	76995	71100	70794	76761	74241	73683
**Pre-consolidation Bulk Density (g/cm^3)**	1.45	1.57	1.53	1.64	1.56	1.54	1.66	1.74	1.67	1.56
**Post-consolidation Volume (mm^3)**	77392.8	79398	72067	73505	74601	69264	69340	73805.4	71948	70997
**Post-consolidation Bulk Density (g/cm^3)**	1.48	1.58	1.55	1.66	1.61	1.58	1.70	1.81	1.72	1.62
**Sample Mass (g)**	114.44	125.72	111.38	122.23	120.01	109.17	117.6	133.33	123.65	115.13
