# Supplementary material for: A bedform phase diagram for dense granular currents
Source: Nat Commun. 2020 Jun 8;11:2873. doi: 10.1038/s41467-020-16657-z (PMC7280512; doi:10.1038/s41467-020-16657-z)
Supplement: Supplementary file 3 — Description of Additional Supplementary Files [file 41467_2020_16657_MOESM3_ESM.pdf]

Description of Additional Supplementary Files

File name: Supplementary Movie 1
Description: Video of an experimental granular current. Deposition is triggered by the transition of the current to an unaerated chamber at 1 m mark (approximately half way across the frame). This is a video of the current seen in Fig. 3.
